# Characterizations and molecular dynamic simulations of broad biologically active arylidene and Quinoxaline cellulose derivatives

**DOI:** 10.1038/s41598-025-14571-2

**Published:** 2025-08-20

**Authors:** Mohamed S. Hasanin, Ahmed A. El-Rashedy, Ahmed K. El-Ziaty, Eslam M. Abbass, Samir Kamel

**Affiliations:** 1 Cellulose & Paper Department, National Research Centre, 33 El-Bohouth St. (Former El-Tahrir St.), Dokki, P.O. 12622, Giza, Egypt; 2 Chemistry of Natural and Microbial Products Department, National Research Center (NRC), Giza, Egypt; 3 Department of Organic and Medicinal Chemistry, Faculty of Pharmacy, University of Sadat City, Sadat City, 32897 Menoufia Egypt; 4 Chemistry Department, Faculty of Science, ASU, P.O. 11566, Cairo, Egypt

**Keywords:** Cellulose tricarboxylate, Anticancer, Antimicrobial, Molecular dynamic simulations, Cyanoacrylate, Arylidines, Quinoxalines, Computational biology and bioinformatics, Materials science

## Abstract

In the current study, oxidized cellulose onto cellulose tricarboxylate (CTC) using 2,2,6,6 tetramethylpiperidine-1-oxyl (TEMPO) and periodate-chlorite oxidation. The ethyl-3-(4-chlorophenyl)-2-cyanoacrylate (W) and 2-chloro3-hydarzinoquinoxaline (R) were formulated into CTC and coded, CTC/W and CTC/R, respectively, that were utilized as ligands in the design synthesis of novel nanocomposites. The prepared nanocomposites were characterized using Fourier-transformed infrared spectroscopy (FTIR), scanning electron microscopy (SEM), and transmission electron microscopy (TEM). Molecular docking and Molecular dynamic (MD) simulations that supported the antimicrobial and cytotoxicity assays were carried out. Physicochemical analysis and topographic studies have affirmed the formulation of nanocomposites. The antimicrobial tests revealed a significant.CTC exhibited more potent activity than CTC/W, indicating its potential as an effective antimicrobial agent. The cytotoxicity test against BJ1 normal cells showed a low effect toward nanocomposites at a 100 µg/mL concentration. A molecular dynamics simulation study of the most active CTC/R and CTC/W was performed to calculate binding free energies using molecular mechanics-generalized born surface area (MM/GBSA). Furthermore, the computational studies revealed that CTC/W showed a high affinity toward the active site of E. coli beta-Ketoacyl-acyl carrier protein synthase III Ec FabH, which provides a strong platform for new structure-based design efforts.

**Supplementary Information:**

The online version contains supplementary material available at 10.1038/s41598-025-14571-2.

## Introduction

Cancer is one of the leading causes of death worldwide^1^. When cancer cells directly extend into nearby tissues and penetrate those tissues, this is referred to as invasion. A break in the tissue barriers eventually results from the growth of altered cells and the gradual expansion of the tumor, allowing the cancer to extend into nearby tissue^2^. Furthermore, microbial secondary infection is usually associated with cancer and cancer treatment as well. Antimicrobial activity mechanisms are classified generally to Antibacterial activity is typically categorized into five mechanisms: disruption of bacterial cell wall construction, inhibition of bacterial protein synthesis, inhibition of bacterial nucleic acid synthesis, disruption of metabolic pathways, and impairment of bacterial membrane function^3–5^. Biomedical researchers’ new strategy is finding new agents characterized by dual-function antimicrobial and anticancer^6^. However, the main drawback of these agents is a lack of biocompatibility^7^. In this context, the potential of formulating these agents into a biopolymer is significant, as it could overcome the disadvantages and enhance the activity, offering a ray of hope in the fight against cancer. The most abundant biopolymer on Earth is cellulose, a promising solution due to its widespread availability and renewable nature. It has been used in different applications such as medicine, the food industry, cosmetics, water treatment, electronic devices, and other applications^8^.

The utilization of nanocellulose in the form of nanofibrils, nanocrystals, or bacterial nanocellulose has recently attracted more fascinating studies. Microfibrils are created when (β-D-glucopyranose) units of cellulose chains are combined via β−1,4-glycosidic linkages. The hydroxyl groups (-OH) on the surface of cellulose fibers’ amorphous structure make it a suitable substrate for chemical functionalization reactions. One of the primary methods for adding carboxyl groups (-COOH) to cellulose matrices and transforming them into derivatives with additional value is oxidation. The primary -OH at C6 is being selectively oxidized to the -COOH using 2,2,6,6-tetramethylpiperidine-1-oxyl (TEMPO) as a mediated oxidizing agent^9^. TEMPO is a water-soluble, commercially available, and stable nitroxyl radical, and its catalytic oxidation has opened a new field of selective conversion of alcoholic hydroxyl groups to carboxyl, ketone, and aldehyde groups under mild conditions with high efficiency^10^. When oxidized, cellulose gains distinctive properties like a high water affinity and improved biodegradability, which overcomes the drawbacks of pristine cellulose. Due to its exceptional biocompatibility, water absorption, and chemical capabilities, cellulose merits special consideration for biomedical applications. For example, folic acid-grafted TEMPO-oxidized cellulose was reported as a doxorubicin carrier system^11^. TEMPO/NaClO/NaClO_2_ oxidized cellulose bead was reported as pH-responsive for indomethacin and fenofibrate release. It was found that the release was faster with a high degree of oxidation, which is attributed to a higher degree of re-swelling and higher hydrophilicity of oxidized cellulose^12^.

**Fig. 1:**
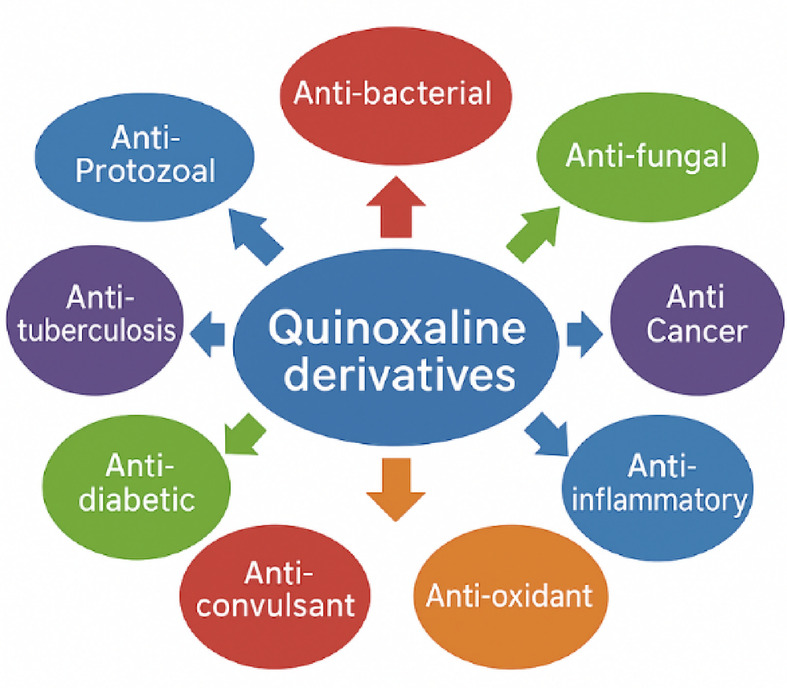
Pharmaceutical applications of quinoxalines.

On the other hand, arylidene compounds are essential, and the chemistry of different arylidene compounds has generated intensive scientific studies worldwide. Arylidene derivatives are of great interest, focused on the synthesis and biological activities of varying arylidene compounds. They are considered building blocks of novel heterocyclic compounds with suitable pharmaceutical activities that can be designed^13^. These arylidenes are usually prepared through the traditional Knoevenagel condensations of aldehydes with active methylene compounds; they are traditionally bases^14^. In addition, quinoxaline derivatives are crucial heterocyclic compounds widely used in medicinal chemistry, and their various derivatives have important biological and medical applications (Fig. 1**)**. The quinoxaline nucleus represents a particular class of pharmacophores observed in multiple natural and synthetic therapeutic agents^15^. Compounds containing quinoxaline core have been found to exhibit several biological activities such as antidepressant, anticancer, antidiabetic, anti-inflammatory, antimicrobial, antiviral, antibacterial, and antifungal activities^16^. Also, quinoxaline derivatives have anti-Protozoal, anti-tuberculosis, anti-convulsant, and antioxidant activities^17^.

In this regard, the present study aims to formulate ethyl-3-(4-chlorophenyl)−2-cyanoacrylate (W) and 2-chloro3-hydarzinoquinoxaline (R) onto cellulose tricarboxylate (CTC) that was prepared by TEMPO, followed by periodate-chlorite oxidation of cellulose based on the selectivity of these oxidizing agents. The confirmation of oxidation and coupling was achieved through FTIR. The study further explores these compounds’ morphology, biological activity, and molecular dynamic simulations.

## Materials and methods

### Materials

Quena Company of Paper Industry, Egypt, supplied bleached bagasse pulp. Its chemical composition was 96% cellulose, 3% hemicellulose, and a shallow lignin content. Sodium metaperiodate (NaIO_4_), sodium bromide (NaBr), sodium hypochlorite (NaClO), sodium chlorite (NaClO_2_), 2,2,6,6-tetramethylpiperidine-1-oxyl (TEMPO), 4-chlorobenzaldehyde, ethyl cyanoacetate, o-phenylenediamine, and oxalic acid were purchased from Sigma Aldrich. All chemical reagents were used without further purification. The microbial and cell line media were purchased from Loba Chem, India.

### Methods

#### Oxidation of cellulose to cellulose tricarboxylate

The cellulose pulp was oxidized to cellulose tricarboxylate through three steps^18^: (a) TEMPO oxidation.

5 g cellulose pulp was dispersed in 500 mL distilled water with 0.08 g TEMPO and 0.8 g NaBr. Then, 50 mL (10%) NaClO was added while stirring, and the pH was adjusted to 10. At the end of the reaction, the pH was adjusted to 7, and the product was collected by centrifugation at 7000 rpm. Further purification was done by dialysis for 1 week against deionized water, which gave TEMPO-oxidized cellulose (carboxy cellulose). The high-shear homogenizer (T-T18 ULTRA TURRAX) disintegrated the oxidized cellulose at 10,000 rpm using a 2% consistency.

(b) Periodate-Chlorite Oxidation.

The TEMPO-oxidized cellulose (12 g) was diluted with water to 1% consistency and 60 °C in a water bath; NaIO_4_ (46 mmol) was added, and the reaction container was covered with aluminum foil to avoid the periodate’s decomposition. After 3 h, the product (dialdehyde carboxy cellulose) was washed with distilled water and filtering in a funnel.

(c) Chlorite Oxidation.

The dialdehyde carboxy cellulose (4.5 g) was diluted with water to 4.5% consistency. An aqueous solution of NaClO_2_ (50 mmol/40 mL of distilled water) was prepared, and 60 mL of 20% acetic acid was added slowly to obtain a yellowish color. This yellowish solution was mixed with suspended dialdehyde carboxy cellulose and stirred at room temperature for 48 h, giving cellulose tricarboxylate. The cellulose tricarboxylate (CTC) was washed with deionized water and filtered, yielding 80–85%, and the carboxyl content was 3 ± 0.3 mmol/g.

#### Preparation of ligands

**Scheme 1:**
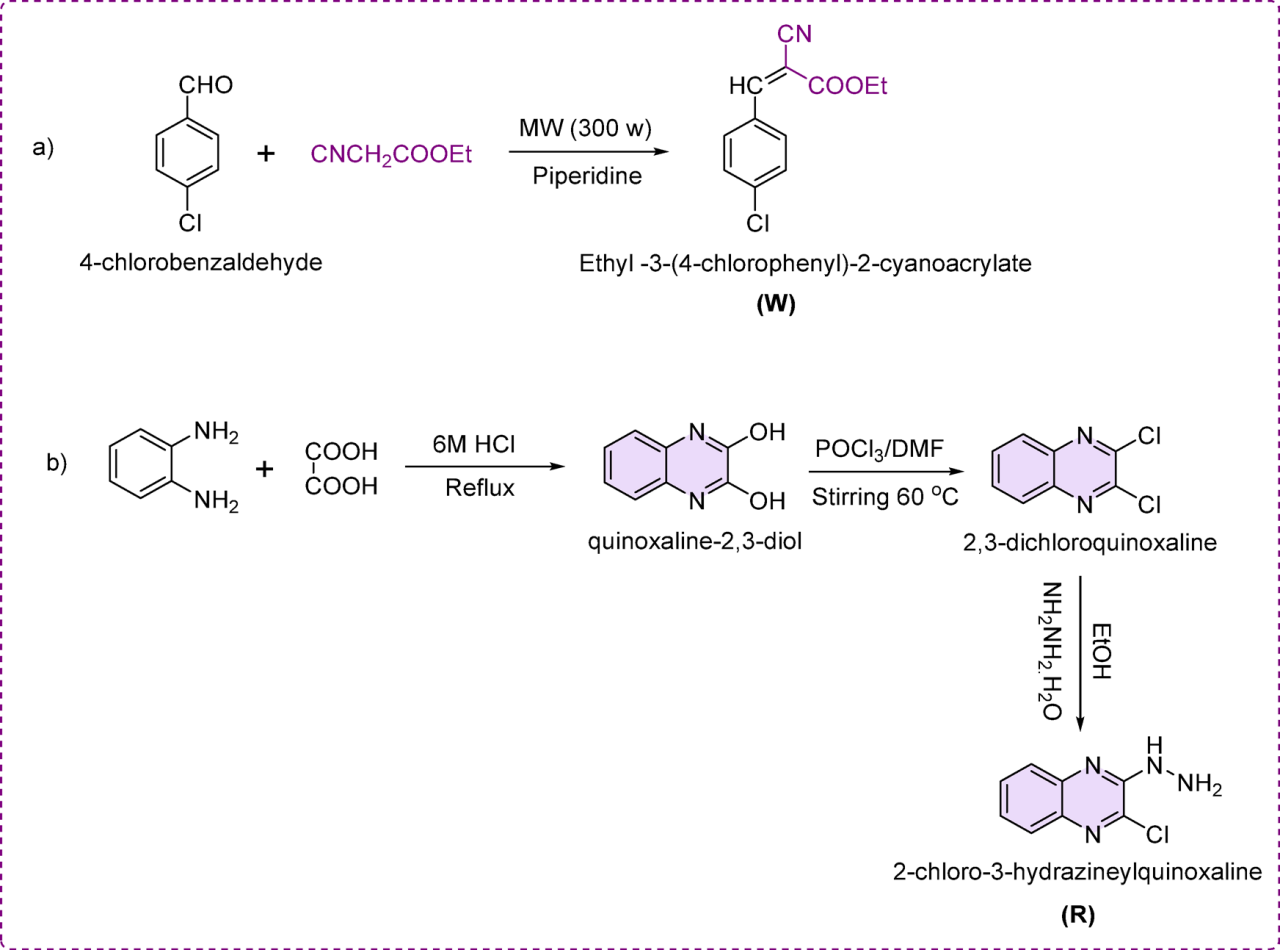
Synthesis of ethyl-3-(4-chlorophenyl)−2-cyanoacrylate and 2-chloro3-hydarzinoquinoxaline.

Both ethyl-3-(4-chlorophenyl)−2-cyanoacrylate and 2-chloro3-hydarzinoquinoxaline were synthesized following literature procedures with some modifications and coded as W and R, respectively (Scheme 1**)**^19^ as follows:

The synthesis of ethyl-3-(4-chlorophenyl)−2-cyanoacrylate was reliably achieved using microwave irradiation in a domestic microwave at 300 W for 1 min. This involved the reaction of 4-chlorobenzaldehyde with ethyl cyanoacetate in the presence of a few drops of piperidine, as illustrated in Scheme 1.

For hydarzinoquinoxaline preparation, the quinoxaline-2,3-diol was initially synthesized by reflux of o-phenylenediamine with oxalic acid in 6 N HCl. The obtained quinoxaline-2,3-diol was stirred with POCl_3_ in dimethylformamide, giving 2,3-dichloroquinoxaline^19^. Finally, the formed 2,3-dichloroquinoxaline was reacted with hydrazine hydrate in ethanol, resulting in nucleophilic substitution and the formation of hydrazinoquinoxaline, as previously reported^19^.

#### Reaction of cellulose tricarboxylate with ligands

The nanocomposites were prepared using ultrasonics and assigned using the microwave method. Aqueous suspension of cellulose tricarboxylate (CTC) (2% w/v) was stirred for 1 h at 70 °C. On the other side, 0.1% (w/v) of W and R was dissolved in Millipore water individually and ultrasonicated using the ultrasonic probe for 3 min in the ice bath. The above-prepared solutions were mixed in equal volumes and stirred at 1500 rpm overnight at 70 °C. The prepared mixtures were microwaved in a domestic microwave for 3 min. The collected nanocomposites and CTC as blanks were lyophilised and maintained in the refrigerator for further use. The prepared nanocomposites were coded as CTC/W and CTC/R related to CTC with ethyl-3-(4-chlorophenyl)−2-cyanoacrylate and 2-chloro3-hydarzinoquinoxaline, respectively.

#### Actual ligand loading efficiency

UV/Vis measurements were conducted with a UV-Vis spectrometer, which was recorded on a Shimadzu model UV-240 spectrophotometer (JASCO, V-730, Japan) in the range of 200–800 nm. UV-vis was carried out with a fixed concentration of ligands. Ligand W was recorded to have a peak at 310 nm, and ligand R was recorded to have a peak at 286 nm (Figure S1). A calibration curve was estimated in a concentration range of 0 to 100 mg/mL for each ligand (Figure S2). On the other side, the concentration of ligands in the nanocomposite was measured at the same wavelength for each ligand^20,21^. Actual ligand loading efficiency was calculated according to the following Eq. (1)^22^.

1 $$ \textrm{Loading} \, \textrm{efficiency} \% = \textrm{M}_{\text{t}}/\textrm{M}_{\text{0}} \times 100 \% $$

Where M_t_ and M_0_ signify the mass of ligand loaded onto the CTC and the initial ligand used, respectively.

#### Characterizations

The characterization of the prepared nanocomposites and comparison with their neat materials were carried out via FTIR spectroscopy (impact-400 FTIR spectrometer) (Nicolet Analytical Instruments, 5225-1, Madison, USA) in the range of 400–4000 cm^−1^ to follow the changes in the functional groups. The topographical analysis was carried out via field emission scanning electron microscopy (FE-SEM) (Republic of Czech Republic, FEI, Quanta FEG 250). The particle’s size and shape were measured using a high-resolution transmission electron microscope (HRTEM) JEOL-JEM-1011, Japan (JEOL 2010, Japan).

### Biological profile of nanocomposites

#### Antimicrobial activity study

The antimicrobial activity of prepared nanocomposites was evaluated using the turbidimetric method as described in our previous work^23,24^ with an initial concentration of 100 µg/mL against bacterial strains, namely *Escherichia coli* ATCC25922, *Staphylococcus aureus* ATCC 25,923, and fungal strains, namely *Candida albicans ATCC90028*. The selected microorganisms were incubated in a nutrient broth medium for 24 h at 37 °C. The fungal strains were grown on potato dextrose broth medium plates and incubated at 30 °C for 3–5 days. The antibiotics streptomycin (standard of a broad-spectrum antibacterial) and griseofulvin (standard of an antifungal) were used as reference drugs. The minimal inhibition concentration (MIC) was evaluated according to our previous work^25^.

#### In vitro study

An in vitro cell line study was conducted and determined by the Bioassay-Cell Culture Laboratory, National Research Centre, Egypt. The following cell lines were used: BJ1 (normal Skin fibroblast), HePG2 (Human hepatocellular carcinoma cell line), HCT116 (Colon cell line), PC3 (Prostate carcinoma cell line), and MCF7 (Human breast carcinoma adenocarcinoma).

#### System Preparation and molecular Docking

The crystal structure of the E. coli beta-Ketoacyl-acyl carrier protein synthase III (*Ec* FabH), solved at a resolution of 1.46Å, was retrieved from the protein data bank with codes 1HNJ and prepared using UCSF Chimera. PROPKA fixed pH and optimized it to 7.5. The extracted 2D structure was drawn using ChemBioDraw Ultra 12.1. The steepest descent approach and MMFF94 force field in Avogadro software were used to optimize the 2D structure for energy minimization. In preparation for docking, hydrogen atoms were removed using UCSF chimera^26^.

#### Molecular Docking

Docking calculations were performed using AutoDock Vina, and Gasteiger partial charges were assigned during docking. The AutoDock atom types were delineated using the AutoDock graphical user interface provided by MGL tools. The grid box was calculated using the following grid parameters: exhaustiveness = 8, x = 10.4915, y = 10, and z = 10 for the center grid, and x = −29.6673, y = 15.6012, and z = −32.0675 for the dimension. Based on their docking energy, the docked conformations were produced in descending order via the Lamarckian genetic algorithm^27^.

#### Molecular dynamic (MD) simulations

The GPU Amber 18 software package was used for all molecular dynamic simulations. The integrated LEAP module was used for protein optimization and explicit solvation, while the AMBER FF14SB force field was used to determine protein properties. After applying a constraint of 100 kcal/mol Å, the systems were minimized for 2500 steps, and then for 1000 steps, they were fully minimized. After that, the systems were heated gradually over 50 ps from 0 to 300 K, maintaining a fixed volume and number of atoms (NVT) and a collision frequency of 1.0 ps-1 with a potential harmonic restraint of 10 kcal/mol Å. The systems were then equilibrated without restraining at a temperature of 300 K at a constant pressure of 1 bar using the Berendsen barostat^28^. This was followed by MD production for 100 ns for each system, in which the SHACK algorithm was used to constrain the bonds of hydrogen atoms.

#### Post-MD analysis

The CPPTRAJ^29^ module of the AMBER18 suite was used to examine the trajectories after they were saved every 1 ps from the MD simulations. All graphs and visualizations were made using Chimera^30^ and the data analysis tool Origin.

#### Thermodynamic calculation

Estimating ligand-binding affinities has proven helpful in applying the Poisson-Boltzmann or generalized Born and surface area continuum solvation (MM/PBSA and MM/GBSA) approach^31^. Within a specified force field, the Protein-Ligand complex molecular simulations utilized by MM/GBSA and MM/PBSA provide accurate statistical-mechanical binding free energy. Average binding free energy over 200 images taken from the 20 ns trajectory. The following representation can be used to estimate the change in binding free energy (ΔG) for each molecular species, including complex, ligand, and receptors, Eqs. (2–6).

2 $$\:\text{s}\text{u}\text{b}-\text{c}\text{o}\text{m}\text{p}\text{l}\text{e}\text{x}\varDelta\:{\text{G}}_{\text{b}\text{i}\text{n}\text{d}}={\text{G}}_{\text{c}\text{o}\text{m}\text{p}\text{l}\text{e}\text{x}}-{\text{G}}_{\text{r}\text{e}\text{c}\text{e}\text{p}\text{t}\text{o}\text{r}}-{\text{G}}_{\text{l}\text{i}\text{g}\text{a}\text{n}\text{d}}\:\:\:\:\:\:\:\:\:\:$$

3 $$\:\varDelta\:{\text{G}}_{\text{b}\text{i}\text{n}\text{d}}={\text{E}}_{\text{g}\text{a}\text{s}}+{\text{G}}_{\text{s}\text{o}\text{l}}-\text{T}\text{S}\:\:\:\:\:\:\:\:\:\:\:\:\:\:\:\:\:\:\:\:\:\:\:\:\:\:\:\:\:\:\:\:\:\:\:\:\:\:\:\:\:\:\:\:\:\:\:\:\:\:\:\:\:\:\:\:\:\:\:\:\:\:\:\:\:\:$$

4 $$\:{\text{E}}_{\text{g}\text{a}\text{s}}={\text{E}}_{\text{i}\text{n}\text{t}}+{\text{E}}_{\text{v}\text{d}\text{w}}+{\text{E}}_{\text{e}\text{l}\text{e}}\:\:\:\:\:\:\:\:\:\:\:\:\:\:\:\:\:\:\:\:\:\:\:\:\:\:\:\:\:\:\:\:\:\:\:\:\:\:\:\:\:\:\:\:\:\:\:\:\:\:\:\:\:\:\:\:\:\:\:\:\:\:\:\:\:\:\:$$

5 $$\:{\text{G}}_{\text{s}\text{o}\text{l}}={\text{G}}_{\text{G}\text{B}}+{\text{G}}_{\text{S}\text{A}}\:\:\:\:\:\:\:\:\:\:\:\:\:\:\:\:\:\:\:\:\:\:\:\:\:\:\:\:\:\:\:\:\:\:\:\:\:\:\:\:\:\:\:\:\:\:\:\:\:\:\:\:\:\:\:\:\:\:\:\:\:\:\:\:\:\:\:\:\:\:\:\:\:\:\:\:\:\:\:\:\:\:\:$$

6 $$\:{\text{G}}_{\text{S}\text{A}}={\upgamma\:}\text{S}\text{A}\text{S}\text{A}\:\:\:\:\:\:\:\:\:\:\:\:\:\:\:\:\:\:\:\:\:\:\:\:\:\:\:\:\:\:\:\:\:\:\:\:\:\:\:\:\:\:\:\:\:\:\:\:\:\:\:\:\:\:\:\:\:\:\:\:\:\:\:\:\:\:\:\:\:\:\:\:\:\:\:\:\:\:\:\:\:\:\:\:\:\:\:\:\:\:\:$$

The terms E_gas_, E_int_, E_ele_, and E_vdw_ represent the gas-phase, internal, Coulomb, and Van Der Waals energy. The FF14SB force field terms were directly used to evaluate the E_gas_. Based on the energy involvement of the polar states (GGB) and non-polar states (G), the calculation of the solution-free energy (Gsol) was performed. Using a water probe radius of 1.4 Å, the non-polar solvation-free energy (GSA) was calculated from the Solvent Accessible Surface Area (SASA)^32^. On the other hand, the contribution of polar solvation was evaluated by solving the GB equation (GGB). The total entropy of the solute and temperature is represented by items S and T, respectively. The contribution of each residue to the total binding free energy was determined using Amber18’s MM/GBSA-binding free energy approach.

## Results and discussions

**Scheme 2:**
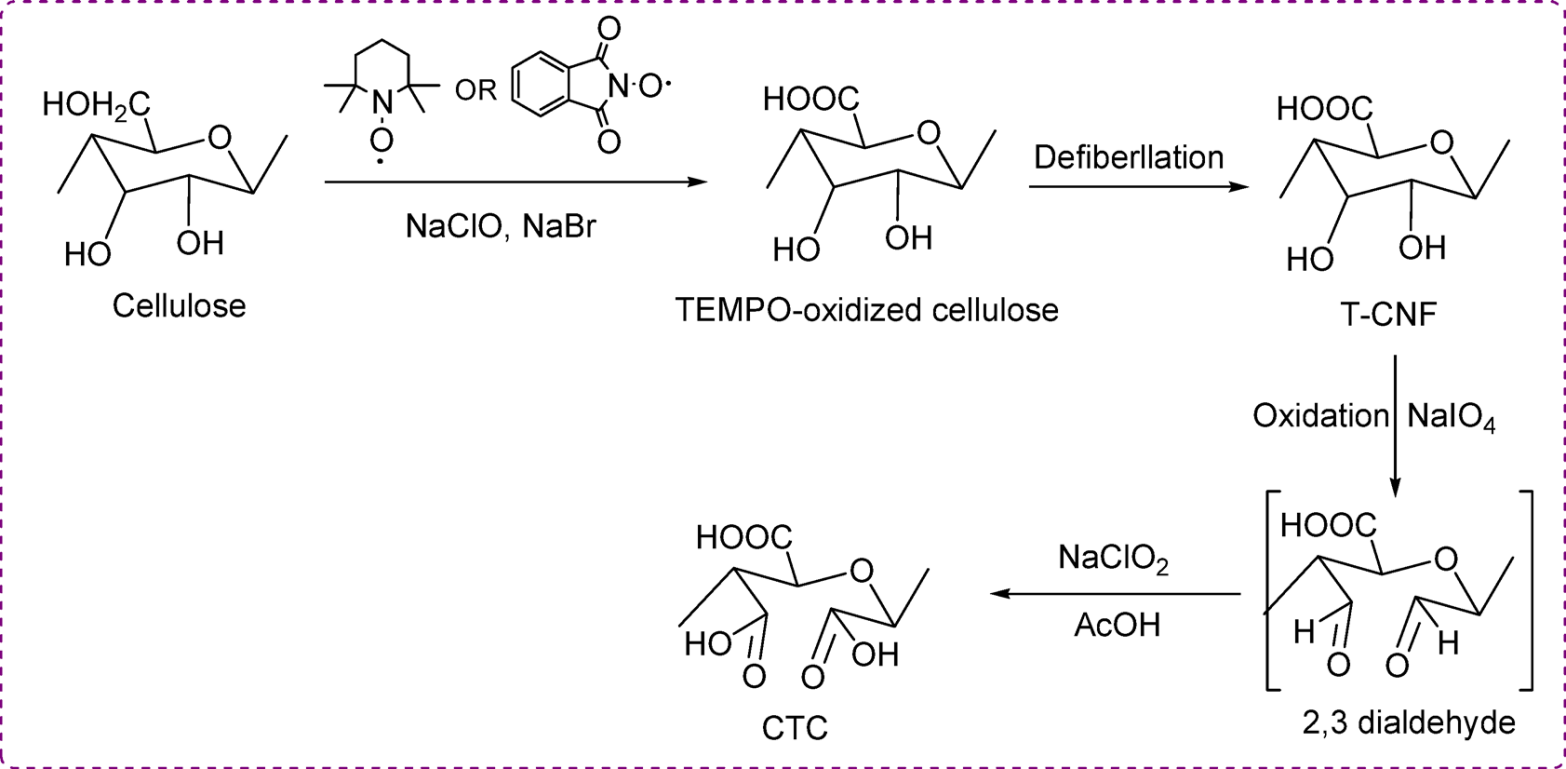
Oxidation of cellulose to CTC.

**Scheme 3:**
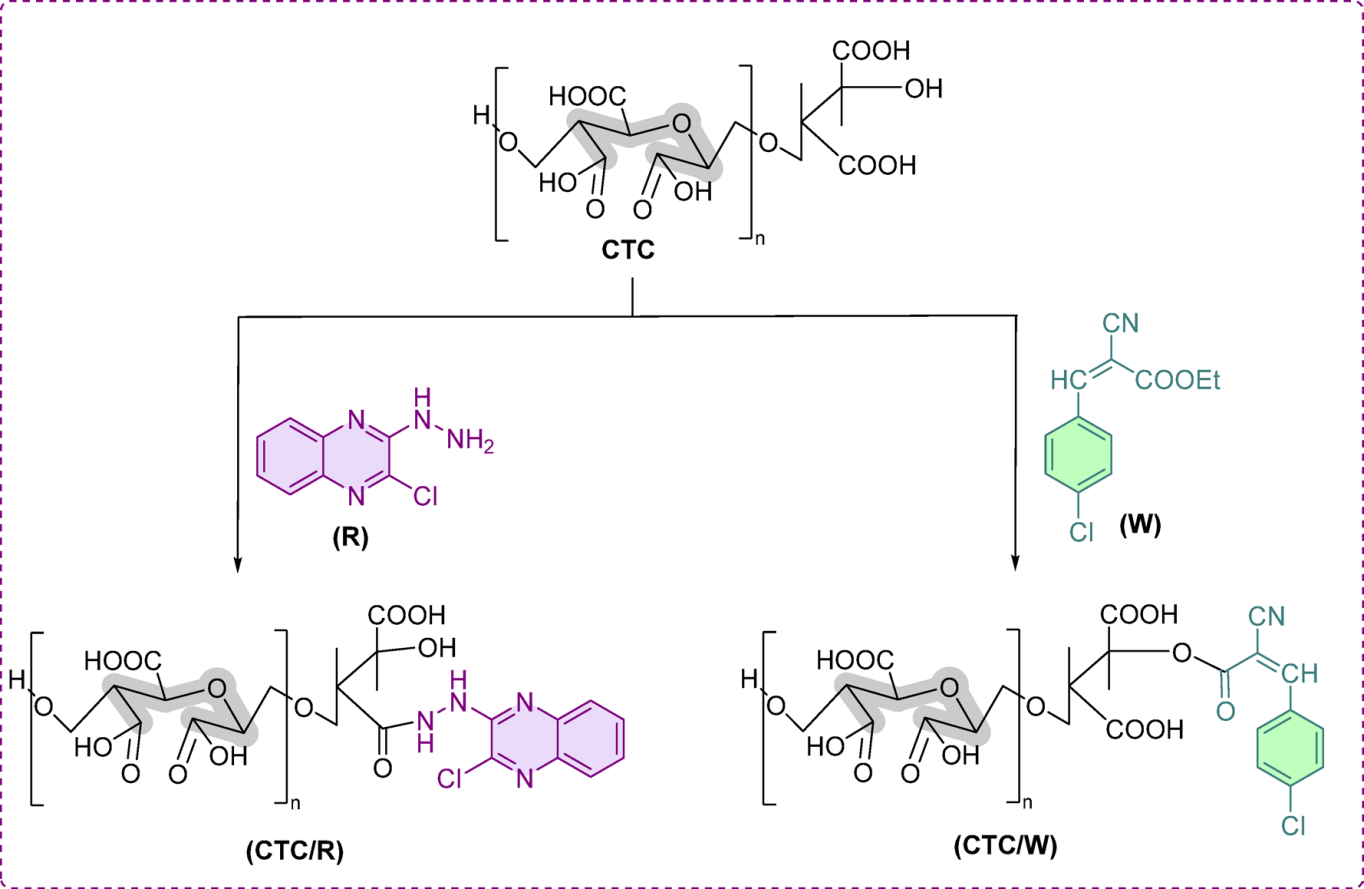
Expected CTC reactions with ligand W and R

**Fig. 2:**
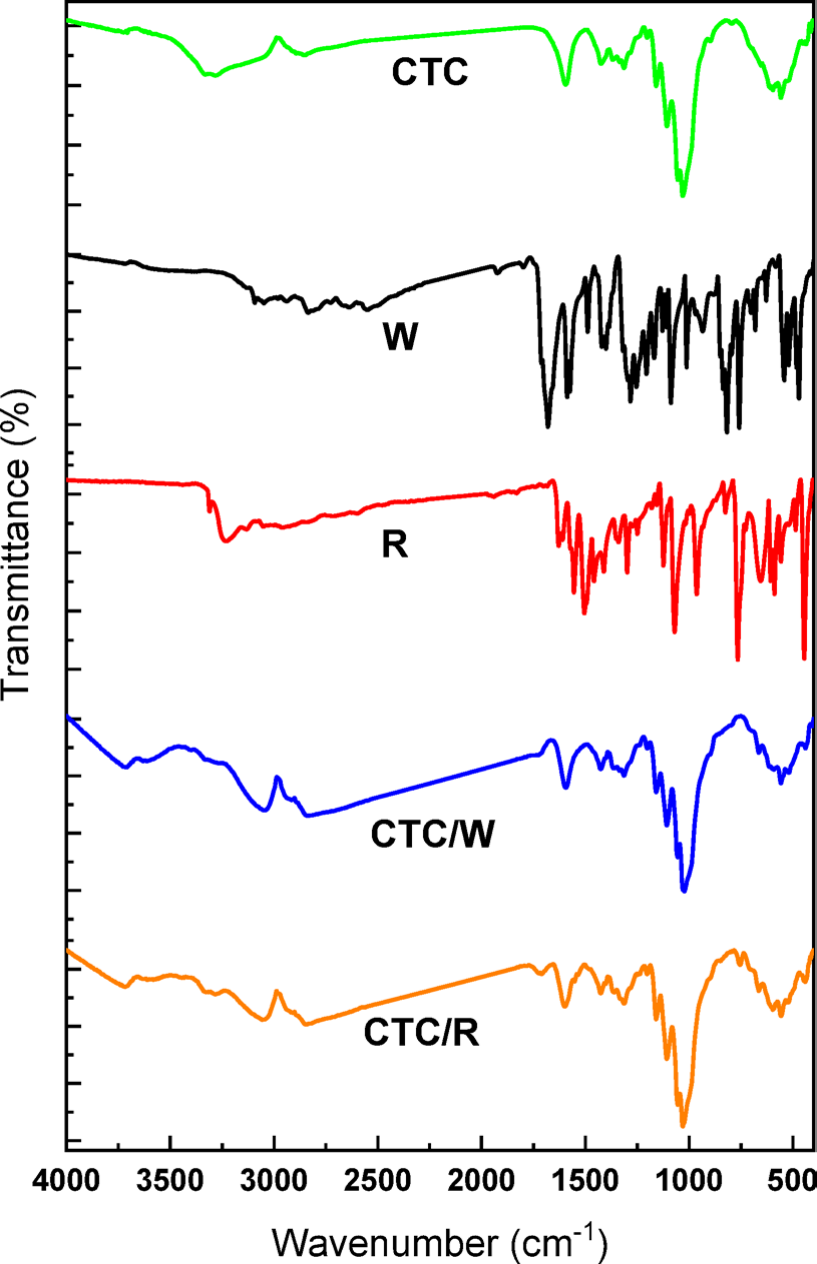
FTIR spectra of CTC, W, R, CTC/W, and CTC/R.

Cellulose tricarboxylate (CTC) was prepared by oxidation of cellulose via TEMPO-mediated reaction, selective at C6, followed by periodate-chlorite oxidation, selective on C2 and C3 (Scheme 2**)**. During this process, the cellulose unit’s primary -OH at C6 was converted into carboxylic acid via the aldehyde stage due to the TEMPO, NaClO, and NaBr oxidation. The secondary -OH at C2 and C3 were initially transformed into aldehydes and further converted to carboxylic acid by the effect of NaIO_4_. The oxidized sample dissolved easily and quickly in the reaction solution due to the availability of -COOH in the cellulose structural unit. The oxidation process was confirmed by the appearance of -COOH at 1600 cm^−1^ in the FTIR-ATR spectra^18^ (Fig. 2). It is expected that the creation of -COOH on the surface of the cellulose facilitates further chemical reactions, such as the coupling of CTC with the prepared ligands (scheme 3). Moreover, the actual ligand loading efficiency for the CTC/W and CTC/R was recorded as 42 ± 3 and 53 ± 4%, respectively.

The FTIR spectra of 2-chloro3-hydarzinoquinoxaline exhibited an absorption band at 3443 cm^−1^, which characterizes the NH group. The absorption of the NH_2_ group is shown at 3310 and 3219 cm^−1^^33^. The 1629 and 1608 cm^−1^ absorption bands characterize the C = N and the aromatic C = C functions. The IR spectra of ethyl-3-(4-chlorophenyl)−2-cyanoacrylate exhibited an absorption band at 3056 cm^−1^, representing the aromatic CH^34^. The absorption of the CN group is shown at 2218 cm^−1^. The absorption bands at 1722 characterize the C = O^35^. The IR spectra of the CTC/W and CTC/R, presented in Fig. 1, compared to those of the prepared ligands and CTC, show that the critical changes are seen mainly in the alcoholic region, and the other portions of the spectrum are little impacted (Fig. 2**)**.

### Topographical analysis

**Fig. 3:**
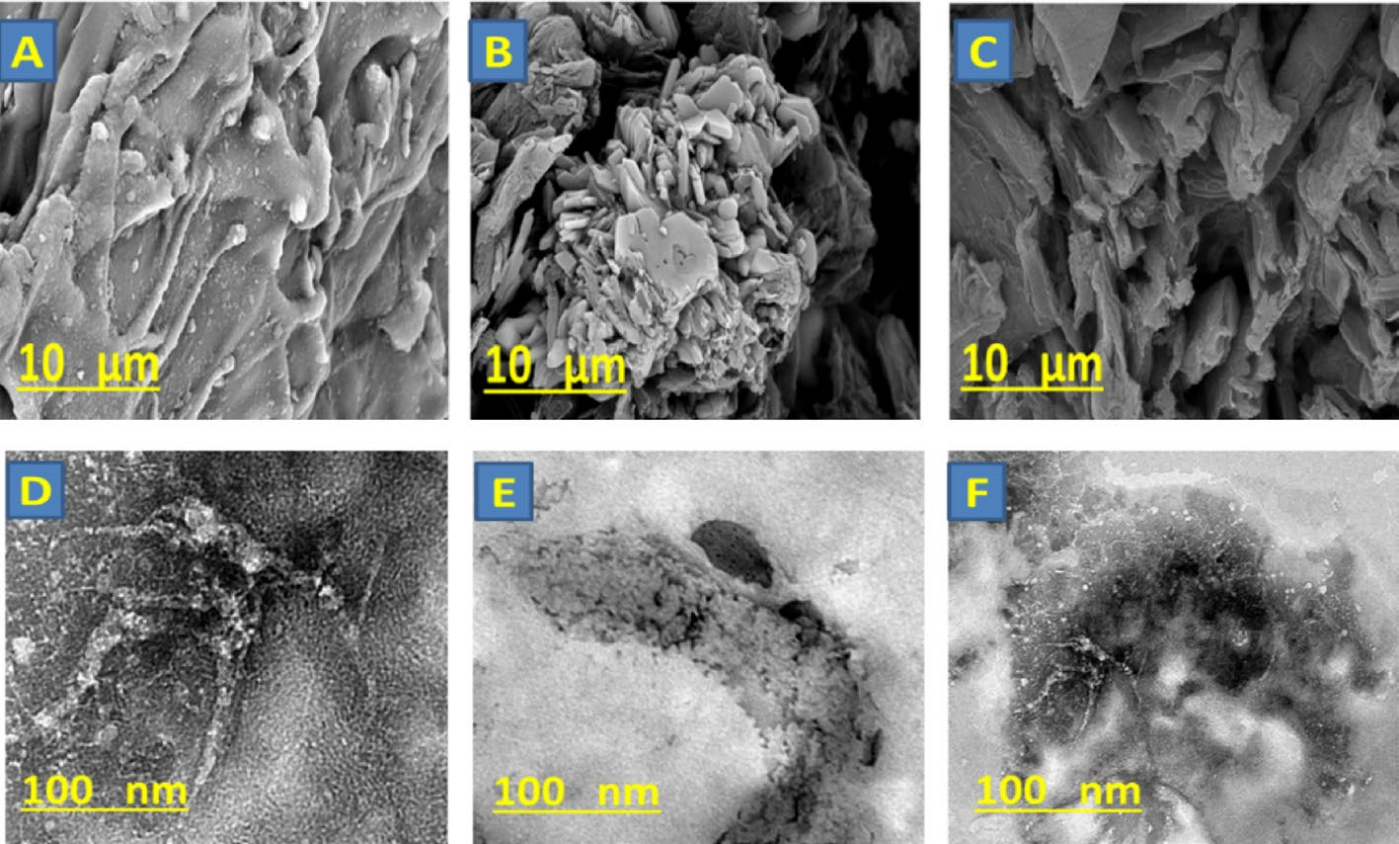
**(A**, **B**, and **C**) are SEM images of CTC, CTC/R, and CTC/W, respectively. (**D**, **E**, and **F**) are TEM images of CTC, R, and W, respectively.

The topographical study, SEM, and TEM images were presented in Fig. 3. The SEM images of CTC (Fig. 3A) showed melted fibers, which referred to the modification of cellulose fibers to CTC. This could result from carboxylate groups that made the fibers stickier. Figure 3 presents the CTC/R with a surface containing granules attached to fibers that convert the CTC surface to rough behaviors. Additionally, the CTC/W (Fig. 3C) surface behaviors were observed as a rough appearance with more homogeneity than the CTC/R surface.

On the other side, the TEM images (Fig. 3) were confirmed by the SEM study, where the CTC (Fig. 3D) was shown as visually striking nanofibers with a diameter of about 9 nm and a length of more than 100 nm. Otherwise, the CTC/R (Fig. 3E) was observed as a fibrinous structure doped with nanoparticles, and the CTC fiber’s appearance was assigned as a rough surface. In addition, the CTC/W (Fig. 3F) was presented as a fibrous behavior structure with some nanoparticles attached over the surface, and the surface was noted to have low roughness compared with CTC/R, providing a visually compelling contrast. These observations affirmed the formulation of the nanocomposites with new features and appearance other than the neat CTC.

### Biological profile

The biological activity of the prepared nanocomposites and CTC was evaluated against some selective pathogenic isolated microbes, including Gram-positive and Gram-negative bacteria, as well as unicellular and filamentous fungi. Additionally, the anticancer activity was carried out against HePG2 cell lines, HCT116 cell lines, PC3 cell lines, and MCF7 cell lines, and a cytocompatibility assay was carried out against BJ1 to complete the picture of the use of the prepared composites with biological systems.

### Antimicrobial analysis

**Fig. 4:**
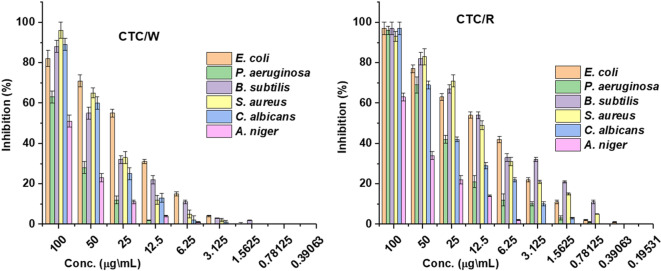
MIC of CTC/R and CTC/W.

The prepared nanocomposites and CTC were subject to tested antimicrobial activity against *B. subtilis*,* S. aureus*,* P. aeruginosa*,* E. coli*,* C. albicans*, and *A. niger* with fixed concentration 100 µg/mL as tabulated in Table 1. The CTC/R has presented a broad antimicrobial spectrum with high inhibition ability, and CTC/W reflected an excellent antimicrobial activity, lower than CTC/R. Otherwise, CTC presented no antimicrobial activity, which was expected according to the lack of active groups that could affect microbial growth. The ligand antimicrobial activity was gained from the nitrogen atom and chlorine involved in the ligand structure^36,37^. In this context, double nitrogen in nanocomposite CTC/R presented a more potent effect against the tested microorganisms with synergetic activity in chlorine^38^. On the other side, the MICs of nanocomposites were presented in Fig. 4 and confirmed that the MIC values were not more than 1.5 and 0.3 µg/mL for CTC/W and CTC/R, respectively, against all microbial populations. These findings affirmed that the nanocomposites have robust antimicrobial activity at low concentrations. Moreover, the CTC/R presented the most potent antimicrobial activity compared to CTC/W. This could be due to nitrogen atoms in CTC/R being three atoms, two in a heterocyclic ring, and the other as a terminal.

**Table 1:** The antimicrobial activity of CTC, CTC/R, and CTC/W.

Tested samples	Microorganisms					
	B. subtilis	S. aureus	*P*. aeruginosa	E. coli	C. albicans	A. niger
**CTC**	0*	0	0	0	0	0
**CTC/R**	97 ± 3	93 ± 4	96 ± 3	97 ± 3	97 ± 4	63 ± 5
**CTC/W**	88 ± 4	96 ± 4	63 ± 3	82 ± 3	89 ± 3	51 ± 4
**Streptomycin**	76 ± 3	84 ± 3	64 ± 5	60 ± 4	NA**	NA
**Griseofulvin**	NA	NA	NA	NA	71 ± 3	55 ± 4

*antimicrobial activity% %.

** This antibiotic does not apply to this strain.

### In vitro study 

**Fig. 5:**
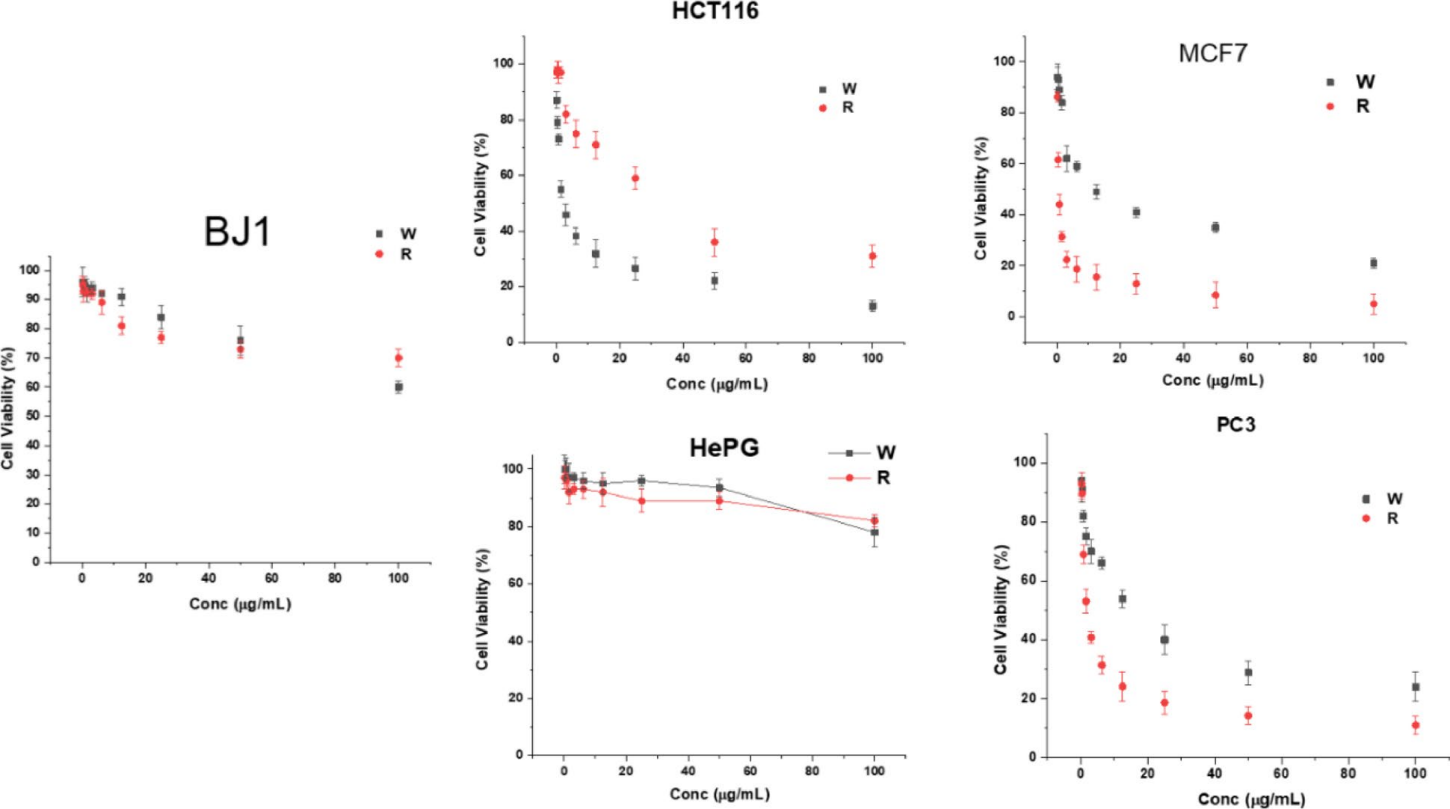
The cell lines assay for prepared CTC/R and CTC/W against normal and cancer cell lines.

The prepared nanocomposites were tested against some selective cell lines to confirm the cytotoxicity and anticancer activity, as shown in Fig. 5. The BJ1 normal cell was shown to have a low effect on nanocomposites at a 100 µg/mL concentration. Moreover, the cell’s growth behavior presented remarks of non-significant changes. On the other hand, the cell line HCT116 presented an effect after treatment with nanocomposites, and the CTC/W presented a more substantial impact than CTC/R. The cell lines MCF7 and PC3 presented typical behavior as well. However, the cell line HePG presented a low effect on towered nanocomposites, which were assigned changes in the cell foliations. These findings emphasized that the prepared nanocomposites could be a strong anticancer agent against HCT116, MCF7, and PC3 with low cytotoxicity.

### Molecular dynamic and system stability

**Fig. 6:**
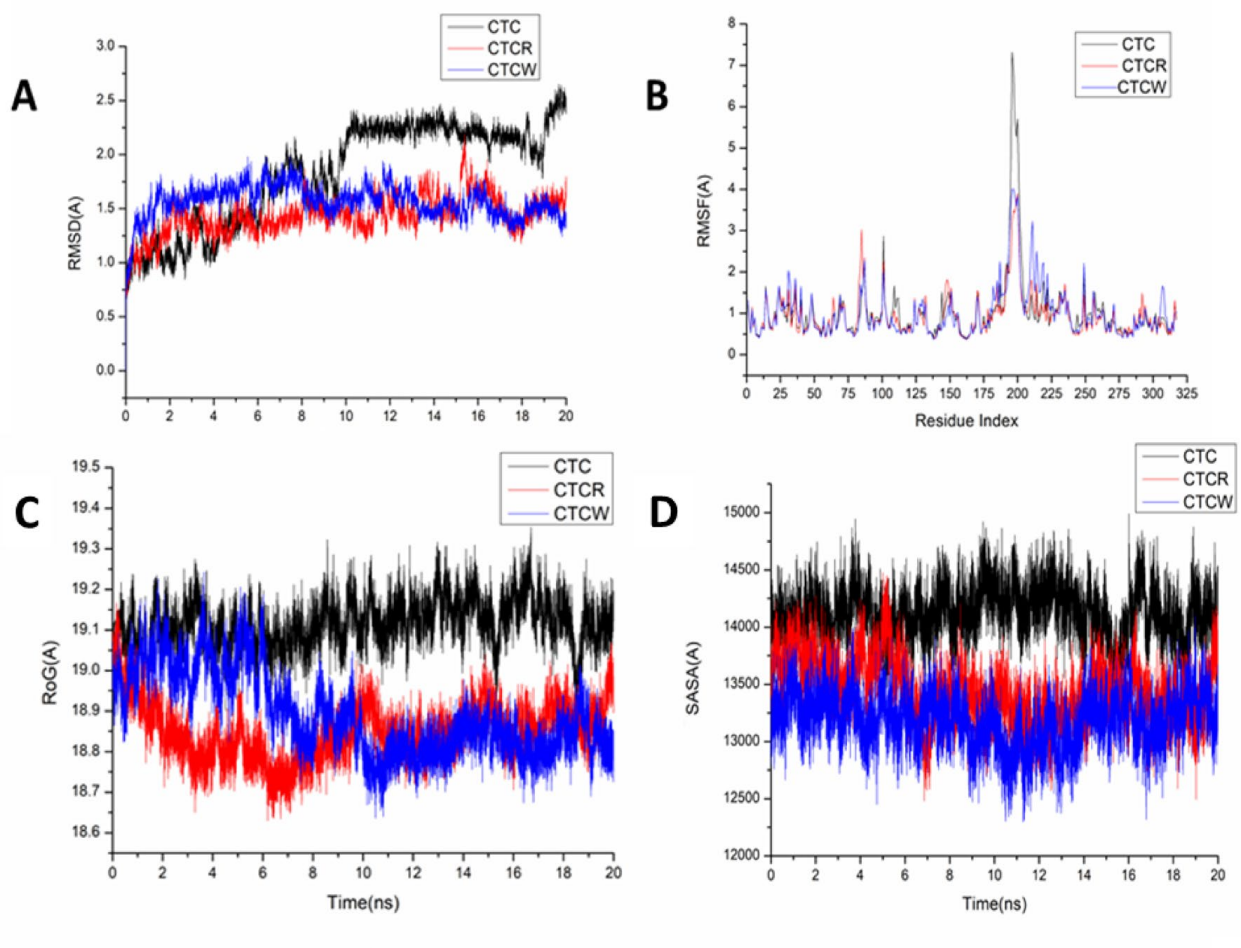
(**A**) RMSD of Cα atoms of the protein backbone atoms, (**B**) RMSF of each residue of the protein backbone Cα atoms of protein residues, (**C**) ROG of Cα atoms of protein residues, and (**D**) SASA of the Cα of the backbone atoms relative (black) to the starting minimized over 20 ns for the catalytic binding site with CTC/R, and CTC/W.

A molecular dynamic simulation was run to forecast the behavior of the prepared compounds upon binding to the protein’s active site, as well as its interaction and stability^39,40^. System stability must be validated to identify interrupted motions and prevent any artifacts during the simulation. The stability of the systems was evaluated in this study using Root-Mean-Square Deviation (RMSD) during the 20 ns simulations. For the apo-protein, CTC/R, and CTC/W systems, the average recorded RMSD values were 1.82 ± 0.47, 1.43 ± 0.18, and 1.56 ± 0.151 Å, in that order (Fig. 6A). These results revealed that the W bound to the protein complex system acquired a relatively more stable conformation than the other studied systems.

Examining residue behavior and its relationship to the ligand during MD simulation requires evaluating the structural flexibility of the protein following ligand binding^41^. Using the Root-Mean-Square Fluctuation (RMSF) technique, protein residue variations were assessed during 20 ns simulations to determine the impact of inhibitor binding on the various targets. For apo-protein, CTC/R, and CTC/W, the computed average RMSF values were 1.03 ± 0.81, 0.944 ± 0.53, and 1.00 ± 0.61Å, respectively. Figure 6B shows the overall residue fluctuations of each system separately. These values revealed that the W bound to the protein complex system has a lower residue fluctuation than the other systems.

During MD simulation, ROG was established to assess the overall compactness and stability of the system upon ligand binding. As shown in Fig. 6C, the average Rg values for the CTC/R system, apo-protein, and CTC/W systems were 19.11 ± 0.05, 18.850 ± 0.07, and 18.890 ± 0.10 Å, respectively. According to the observed behavior, the W-bound complex has a highly stiff structure against the catalytic binding site of the target receptor.

The compactness of the protein hydrophobic core was examined by calculating the protein’s solvent-accessible surface area (SASA). This was performed by measuring the surface area of the protein visible to the solvent, which is essential for biomolecule stability. The average SASA values were 14118.61, 13180.48, and 13434.87 Å for Apo-protein, CTC/R, and CTC/W, respectively (Fig. 6D). The SASA finding, when paired with the observations from the RMSD, RMSF, and ROG computations, confirmed that the CTC/W complex system remains intact inside the kelch domain binding site of the target receptor.

### The binding interaction mechanism based on binding free energy calculation

The molecular mechanics energy methodology (MM/GBSA), which combines the generalized born and surface area continuum solvation, is a prominent method for measuring the free binding energies of small molecules to biological macromolecules and may be more reliable than docking scores^42^. By collecting snapshots from the trajectories of the systems, the binding free energies were computed using the MM-GBSA tool in AMBER18. Table 2 displays that, except for ΔGsolv, every reported estimated energy component had high negative values, indicating positive interactions. The findings showed that the CTC/R and CTC/W had binding affinities of −33.21 and − 30.29 kcal/mol, respectively.

**Table 2:** Calculated energy binding for CTC/R and CTC/W against the catalytic binding site of the FabH receptor.

Energy Components (kcal/mol)					
Nanocomposites	ΔE_vdW_	ΔE_elec_	ΔG_gas_	ΔG_solv_	ΔG_bind_
CTC/R	−50.9073 ± 0.31	−35.0905 ± 1.04	−85.997 ± 0.94	52.78 ± 0.76	−33.21 ± 0.366
CTC/W	−48.0079 ± 0.35	−16.228 ± 0.64	−64.23 ± 0.58	33.94 ± 0.58	−30.29 ± 0.36

∆EvdW = van der Waals energy; ∆Eele = electrostatic energy; ∆Gsolv = solvation free energy; ∆Gbind = calculated total binding free energy.

The interactions between the ligand compounds and the *E. coli* beta-Ketoacyl-acyl carrier protein synthase III (FabH) protein receptor residues are driven by the more positive Van der Waals energy component, as shown by a detailed examination of each energy contribution, leading to the reported binding free energies. Substantial binding free energy values were observed in the gas phase for all the inhibition processes, with values up to −149.227 kcal/mol (Table 2).

### Identification of the critical residues responsible for ligand binding

**Fig. 7:**
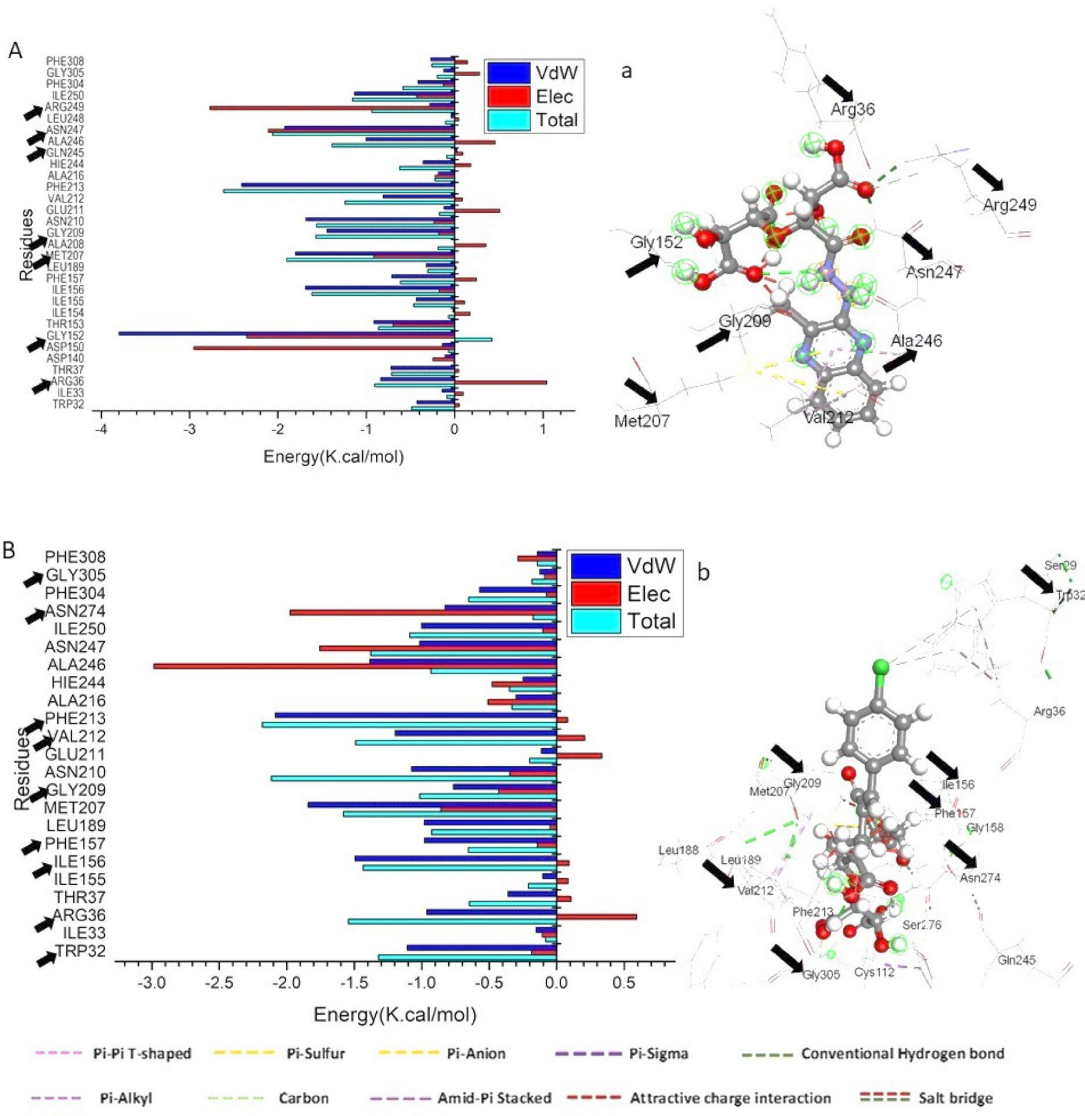
Per-residue decomposition plots showing the energy contributions to the binding and stabilization of CTC/W [**A**] and CTC/R [**B**] into the catalytic binding site of *Ec*FabH, Corresponding inter-molecular interactions are shown (a and b). In contrast, the black arrow indicates ATP binding site interaction residues.

The total energy involved when the compound binds these enzymes was further decomposed into the involvement of individual site residues to get more knowledge about essential residues involved in the inhibition of the catalytic binding site of the FabH receptor. From Fig. 7A, the significant favorable contribution of CTC/R to the FabH binding site receptor is predominantly observed from residues Trp 32 (−0.426 kcal/mol), Arg 36 (−0.832 kcal/mol), Thr37 (−0.723 kcal/mol), Gly152(−3.797 kcal/mol), Thr 153 (−0.914 kcal/mol), Ile 155 (−0.436 kcal/mol), Ile 156 (−1.689 kcal/mol), Phe 157 (−0.71 kcal/mol), Met 207 (−1.801 kcal/mol), Gly209 (−1.445 kcal/mol), Asn 210 (−1.684 kcal/mol), Val 212 (−0.811 kcal/mol), Phe 213 (−2.413 kcal/mol), Hie 244 (−0.357 kcal/mol), Ala246 (−1.003 kcal/mol), Asn 247 (−1.923 kcal/mol), Ile 250 (−1.134 kcal/mol), and Phe 304 (−0.414 kcal/mol).

On the other hand, the significant favorable contribution of CTC/W to the FabH binding site receptor is predominantly observed from residues Trp 32 (−1.107 kcal/mol), Arg 36 (−0.963 kcal/mol), Thr37 (−0.361 kcal/mol), Ile 156 (−1.495 kcal/mol), Phe 157 (−0.979 kcal/mol), Leu189 (−0.98 kcal/mol), Met 207 (−1.8421 kcal/mol), Gly209 (−0.764 kcal/mol), Asn 210 (−1.076 kcal/mol), Val 212 (−1.197 kcal/mol),), Phe 213 (−2.085 kcal/mol), Ala216 (−0.30 kcal/mol), Hie 244 (−0.248 kcal/mol), Ala246 (−1.385 kcal/mol), Asn 247 (−1.017 kcal/mol), Ile 250 (−1.002 kcal/mol), Asn274 (−0.827 kcal/mol), and Phe 304 (−0.571 kcal/mol), (Fig. 7B).

### Ligand–residue interaction network profiles

Among the molecular targets in pathogenic strains, β-ketoacyl-acyl carrier protein synthase III (FabH), which catalyzes the initial condensation step between acetyl-CoA and malonyl-ACP (30), has been verified as an attractive therapeutic target^43^. Furthermore, FabH is not only highly conserved at the sequence and structural levels among major pathogens, but the amino acid residues constituting its catalytic triad (i.e., Cys-His-Asn) are essentially invariant across Gram-positive and Gram-negative species^44^.

**Fig. 8:**
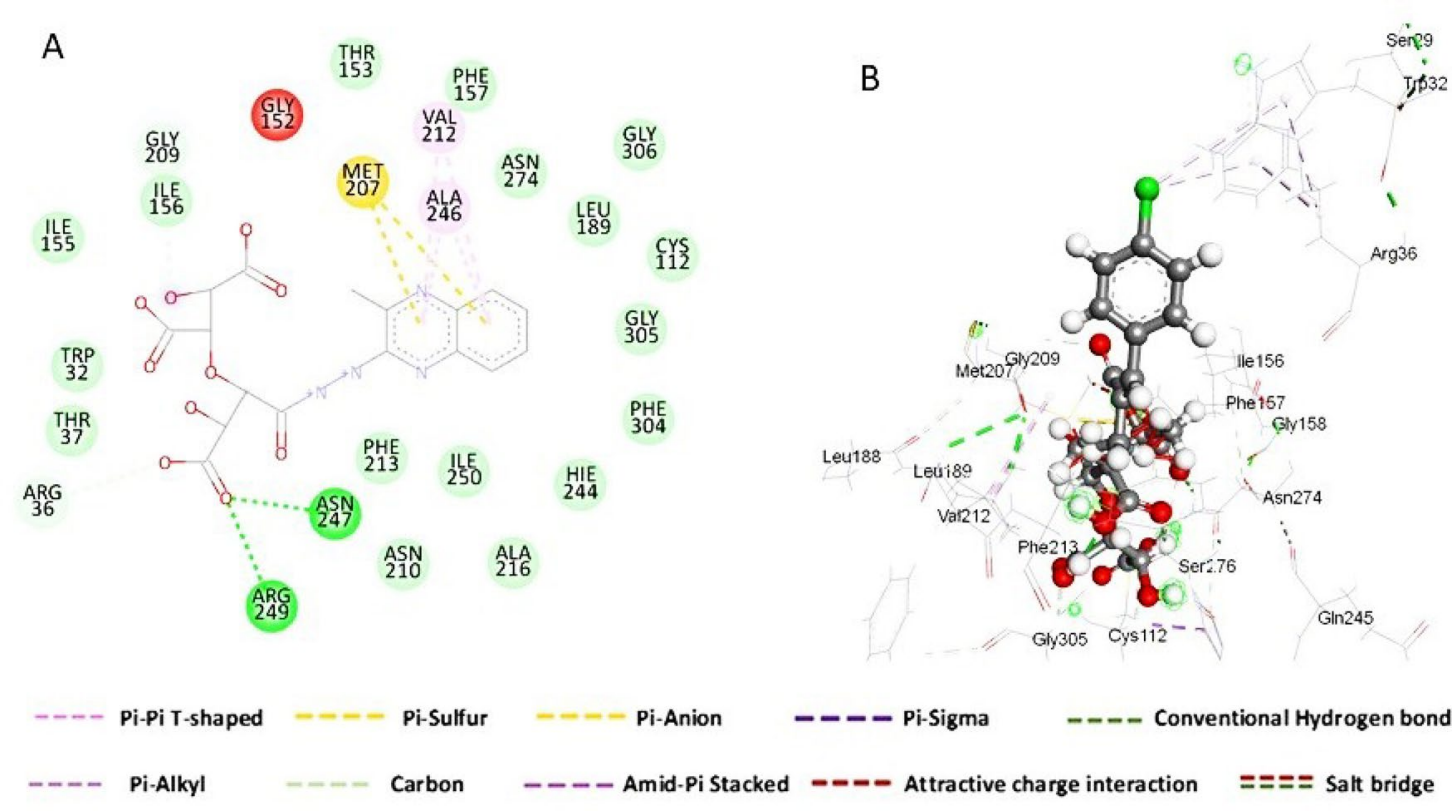
The interaction residue of hydrazinoquinoxaline (**A**) and cyanoacrylate (**B**). into the catalytic binding site of *Ec*FabH.

The hydrazinoquinoxaline (CTC/R) was found near the right entrance of the catalytic cleft, and the hydroxyl group of the cellulose ring formed a secure network of H-bonds with Asn 247 and Asn 249. The bulky group 2-chloroquinoxaline is involved in pi - pi -stacking interactions with Val 212 and Ala 246. Furthermore, the hotspot 2-chloroquinoxaline has formed a pi-anion connection with Met 207 (electrostatic interaction). (Fig. 8A**).**

The hydroxyl group of the cellulose in cyanoacrylate (CTC/W) (Fig. 8B**)**, on the other hand, filled the catalytic pocket via a secure network of H-bonds with Asn 247. The p-chloro phenyl ring has made a pi-alkyl contact with Trp 32 (electrostatic interaction). Furthermore, carbon interactions between the oxygen atom of the cellulose ring and Gly 209 and Gly 305 were illustrated. Finally, the Pi-sulfur interaction between the hydroxyl group of the cellulose ring and Met 207 was investigated.

Such binding mechanisms for both CTC/R and CTC/W imply that both of these compounds may be able to block (or at least partially) substrate access into the *Ec*FabH protein’s active site, which could restrict enzyme activity.

### SAR (structure-activity relationship) studies

From the antimicrobial activity results of the newly synthesized compound, the presence of cyanoacrylate substitution on the cellulose, which has electron-withdrawing groups that make anionic interaction with Trp 32, Gly 209, Gly 305, and Met 207 and showed a remarkable activity compared to hydrazinoquinoxaline substitution, which has hydrazine substitution that acts as a donating group. From these results, an electron-withdrawing group is required for a compound to be effective as an antimicrobial agent.

## Conclusion

For the first time, we report the formulation of Ethyl-3-(4-chlorophenyl)−2-cyanoacrylate 3 and 2-chloro3-hydarzinoquinoxaline 7 on cellulose tricarboxylate. This innovative approach aims to develop novel broad-antimicrobial active nanocomposites based on cellulose. After formulation onto cellulose tricarboxylate, our in vitro study confirmed a significant reduction in arylidene and quinoxaline compounds’ toxicity and side effects. The analysis and topographic studies underscore the formulation of nanocomposites on the nanoscale and the loading of the arylidene and quinoxaline compounds (individually) into the cellulose chain helix. The prepared nanocomposites exhibit clarified cytocompatibility and strong antimicrobial activity, particularly the CTC/R nanocomposite, which shows superior MIC values compared to CTC/W, with approximately the same biocompatibility and anticancer activity. Molecular modeling and MD simulations demonstrate the potential of the cyanoacrylate complex to bind to active sites of the DNA gyrase receptor, suggesting promising applications in antimicrobial and anticancer treatments.

## Supplementary Information

Below is the link to the electronic supplementary material.

Supplementary Material 1

## Data Availability

The datasets used and/or analyzed during the current study are available from the corresponding author on reasonable request.
