# Supplementary material for: Characterizations and molecular dynamic simulations of broad biologically active arylidene and Quinoxaline cellulose derivatives
Source: Sci Rep. 2025 Aug 20;15:30561. doi: 10.1038/s41598-025-14571-2 (PMC12368164; doi:10.1038/s41598-025-14571-2)
Supplement: Supplementary file 1 — Supplementary Material 1 [file 41598_2025_14571_MOESM1_ESM.docx]

**Characterizations and molecular dynamic simulations of broad biologically active arylidene and quinoxaline cellulose derivatives**

Mohamed S. Hasanin^1*^, Ahmed A. El-Rashedy^2^, Ahmed El-Ziaty^3^, Eslam M. Abbass^3^ and Samir Kamel^1^

^1^Cellulose & Paper Department, National Research Centre, 33 El-Bohouth St. (Former El-Tahrir St.), Dokki, Giza, P.O. 12622, Egypt

^2^ Chemistry of Natural and Microbial Products Department, National Research Center (NRC), Egypt

^2^ Department of Organic and Medicinal Chemistry, Faculty of Pharmacy, University of Sadat City, Menoufia, 32897, Egypt

^3^Chemistry Department, Faculty of Science, ASU, Cairo, P.O. 11566, Egypt

Corresponding author: Mohamed S. Hasanin, email: [sido_sci@yahoo.com](mailto:sido_sci@yahoo.com)

|  |
| --- |
| **Figure (1S):** UV-vis spectra of the ligand R and W. |

| **[a]** |  |
| --- | --- |
| **[b]** |  |
|  | **Figure (2S):** Standard curve of the ligand R (a) and W (b). |
